# When a Step Is Not a Step! Specificity Analysis of Five Physical Activity Monitors

**DOI:** 10.1371/journal.pone.0169616

**Published:** 2017-01-13

**Authors:** Sandra O’Connell, Gearóid ÓLaighin, Leo R. Quinlan

**Affiliations:** 1 Discipline of Physiology, School of Medicine, NUI Galway, Galway, Ireland; 2 Electrical & Electronic Engineering, School of Engineering & Informatics, NUI Galway, Galway, Ireland; 3 National Centre for Biomedical Engineering Science, NUI Galway, Galway, Ireland; 4 CÚRAM – Centre for Research in Medical Devices, NUI Galway, Galway, Ireland; Victoria University, AUSTRALIA

## Abstract

**Introduction:**

Physical activity is an essential aspect of a healthy lifestyle for both physical and mental health states. As step count is one of the most utilized measures for quantifying physical activity it is important that activity-monitoring devices be both sensitive and specific in recording actual steps taken and disregard non-stepping body movements. The objective of this study was to assess the specificity of five activity monitors during a variety of prescribed non-stepping activities.

**Methods:**

Participants wore five activity monitors simultaneously for a variety of prescribed activities including deskwork, taking an elevator, taking a bus journey, automobile driving, washing and drying dishes; functional reaching task; indoor cycling; outdoor cycling; and indoor rowing. Each task was carried out for either a specific duration of time or over a specific distance. Activity monitors tested were the ActivPAL micro^™^, NL-2000^™^ pedometer, Withings Smart Activity Monitor Tracker (Pulse O_2_)^™^, Fitbit One^™^ and Jawbone UP^™^. Participants were video-recorded while carrying out the prescribed activities and the false positive step count registered on each activity monitor was obtained and compared to the video.

**Results:**

All activity monitors registered a significant number of false positive steps per minute during one or more of the prescribed activities. The Withings^™^ activity performed best, registering a significant number of false positive steps per minute during the outdoor cycling activity only (P = 0.025). The Jawbone^™^ registered a significant number of false positive steps during the functional reaching task and while washing and drying dishes, which involved arm and hand movement (P < 0.01 for both). The ActivPAL^™^ registered a significant number of false positive steps during the cycling exercises (P < 0.001 for both).

**Conclusion:**

As a number of false positive steps were registered on the activity monitors during the non-stepping activities, the authors conclude that non-stepping physical activities can result in the false detection of steps. This can negatively affect the quantification of physical activity with regard to step count as an output. The Withings^™^ activity monitor performed best with regard to specificity during the activities of daily living tested.

## Introduction

The benefits to general health of physical activity are universally recognized [1–3]. Clear guidelines have been published by the US Surgeon General (1996), the Centers for Disease Control (CDC, 2008) and the World Health Organization (WHO, 2010). To maximize the health benefits of physical activity for the general population, these guidelines recommend that activities, such as brisk walking, be carried out daily [4–6]. An essential part of achieving a behavioral change in promoting physical activity is the capacity to easily monitor goal setting. The use of physical activity monitors has exploded in the last 5 years, both in the consumer market and increasingly as tools for clinical settings [7–9]. The most commonly utilised output parameter from these monitors is the cumulative step count over a day, week and beyond. In line with the WHO recommendations, many individuals aim to improve on their physical activity levels by reaching a personal goal or the recommended goal of 10,000 steps per day [4–6]. Utilizing an easily quantifiable output such as step count supports a person in achieving physical activity goals and maintaining a healthy lifestyle [10, 11]. Cumulative logging of step count can become a key factor in the promotion and maintenance of physical activity levels and mental wellbeing. As persons become increasingly dependent on the activity monitor to unobtrusively record their activity level, the question of the device’s sensitivity and specificity to the reported output becomes an increasingly important one.

We have previously examined the step count sensitivity of a number of physical activity-monitoring devices during walking over a variety of surface types while achieving the recommended daily step goal of 10,000 steps. We found all devices tested to be sensitive in step detection, with a mean absolute percentage error ranging from 1.36% to 4.61% for the four activity monitors tested [12]. However, while this is a very important factor to consider in assessing the performance of an activity monitor, it is equally critical that the activity-monitors are not just sensitive but also specific in their capacity to monitor actual steps performed. The ideal activity monitor should only record a step when a step is actually taken, while disregarding non-stepping body movements.

The literature is sparse when reporting on the specificity of physical activity monitors examined. Chen *et al* [13] investigated three consumer wristband activity monitors; Fitbit Flex, Garmin Vivofit and Jawbone UP during a range of normal daily activities, including playing a tablet computer game, folding laundry, pushing a stroller and carrying a bag. They reported substantial false step detection events during the task of folding laundry and reported the accuracy under each activity to range widely both between monitors and activities performed [13]. In a similar study, Sellers *et al* [14] investigated the ActivPAL3 monitor during a range of activities of daily living. These activities included hanging laundry out to dry, putting on a duvet cover and pillowcase, cleaning a framed picture, writing a letter/list and vacuuming. The authors reported step detection sensitivity of 76.1% for young people but only 40.4% for older adults during normal activities of daily living. While they reported the ActivPAL3 to accurately detect ‘purposeful stepping’, the detection of smaller stepping movements during the activities of daily living was reported as being poor [14]. These studies reveal a significant degree of variability in the step detection specificity during activities that are not solely purposeful stepping activities like walking. Normal activities of daily living include a range of stepping and non-stepping activities and also range from low to high levels of intensity. As step count is now a pervasive and easily understandable measure of physical activity it is essential that monitors be both sensitive and specific in their measures.

The aim of this paper is to assess five activity monitors in healthy adults: the Jawbone UP^™^, Withings Smart Activity Monitor Tracker (Pulse O_2_)^™^, NL-2000 pedometer^™^, ActivPAL micro^™^ and Fitbit One^™^ for their step detection specificity over a range of different physical activities that mimic typical activities of daily living of various intensity levels.

## Methods

### Participants

A pool of 37 participants (12 male) took part in the study involving the performance of 9 prescribed activities which may trigger false detection of stepping: (i) deskwork, (ii) taking an elevator, (iii) taking a bus journey, (iv) driving an automobile, (v) washing and drying dishes, (vi) functional reaching task, (vii) indoor cycling, (viii) outdoor cycling and (iv) indoor rowing. Participants had a mean age of 39 ± 13.87 years and a mean body mass index (BMI) of 25 ± 3.79 kg/m^2^. A random sample of ten participants carried out each activity. The mean ages and BMIs of the ten participants that carried out each activity are given in Table 1. None of the participants had any history of cardiovascular disease or neurological disorders. Ethics committee approval (reference number CA-1069) was obtained from the Galway University Hospitals Research Ethics Committee for this study and all participants provided written, informed consent.

**Table 1:** Demographic details for the ten participants that carried out each specificity activity.

Specificity Activity	Gender	Age (years)	Weight (kg)	BMI (kg/m^2^)
Deskwork	6 female; 4 male	33 ± 11.15	71.28 ± 14.07	24.62 ± 3.96
Elevator	9 female; 1 male	35 ± 14.11	68.40 ± 12.08	24.41 ± 3.98
Bus journey	8 female; 2 male	38 ± 13.91	65.90 ± 13.93	24.09 ± 5.08
Automobile driving	7 female; 3 male	43 ± 14.98	71.06 ± 12.79	25.10 ± 4.88
Washing and drying dishes	9 female; 1 male	32 ± 12.5	63.90 ± 8.52	23.25 ± 3.43
Functional reaching task	9 female; 1 male	32 ± 12.63	66.40 ± 9.25	24.05 ± 3.39
Indoor cycling	9 female; 1 male	32 ± 12.63	64.10 ± 10.72	23.22 ± 3.42
Outdoor cycling	8 female; 2 male	29 ± 8.68	65.56 ± 10.13	23.92 ± 3.08
Indoor Rowing	7 female; 3 male	48 ± 10.61	71.28 ± 13.55	23.86 ± 3.11

All values represent mean ± SD. BMI = Body Mass Index; SD = Standard Deviation.

### Study protocol

**Fig 1:**
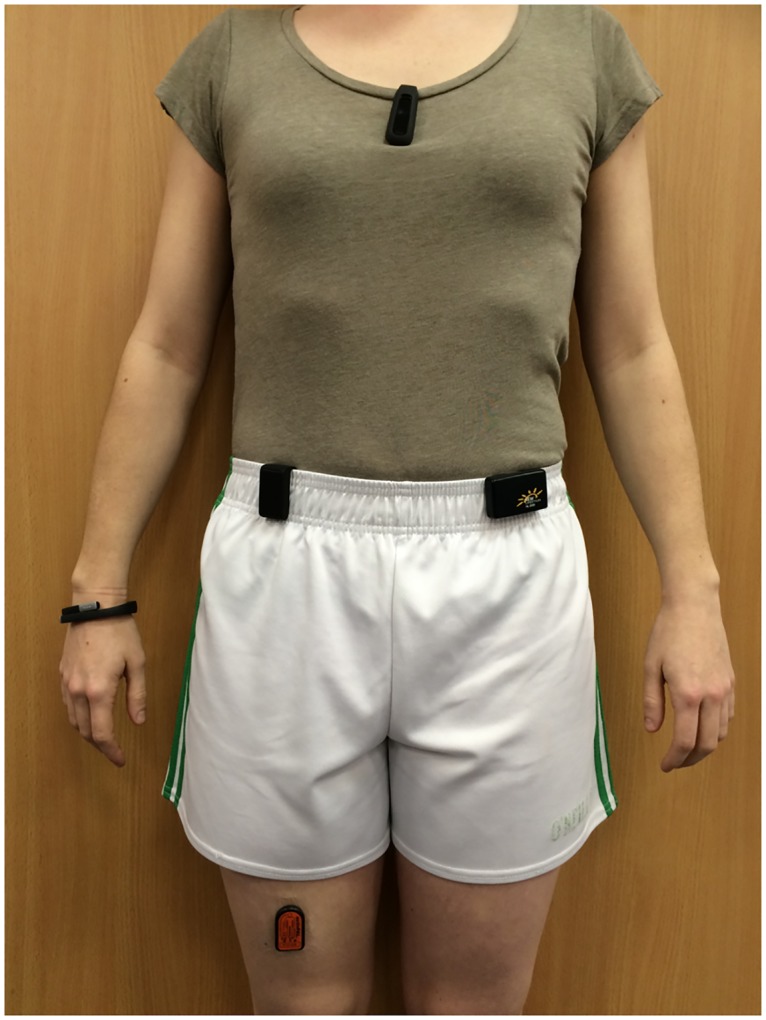
Positioning of each activity monitor. The ActivPAL^™^ is worn on the right thigh, the NL-2000^™^ on the left hip, the Withings^™^ on the right hip, the Jawbone^™^ on the right wrist and the Fitbit^™^ at the level of the chest.

All activity monitors were positioned as per manufacturer’s recommended body locations (Fig 1). Tegaderm transparent dressing (3M Health Care, Minnesota, USA) was used to affix the ActivPAL^™^ to the mid-anterior aspect of the thigh, with the Jawbone UP^™^ worn on the wrist, the Withings Smart Activity Monitor Tracker (Pulse O_2_)^™^ and NL-2000 pedometer^™^ worn on opposite hips and the Fitbit One^™^ clipped on at the level of the chest.

Participants wore the activity monitors simultaneously for the duration of each test condition. For the NL-2000^™^, Withings^™^ and Fitbit^™^ monitors the recorded step count was obtained directly from the device screen interface. To extract the step count data from the Jawbone^™^, it was synced to a smartphone via Bluetooth and step count was obtained from the Jawbone^™^ smartphone application. To extract the step count data from the ActivPAL^™^, it was manually connected to a PC and the data downloaded using the ActivPAL^™^ software program, which outputs the step count data.

To ensure the step count was accurate for the activity only, and not contaminated by activity just before or after the prescribed activity, specific procedures were put in place for each monitor. For the NL-2000^™^, Withings^™^ and Fitbit^™^ monitors, the step counts displayed on the device interfaces were noted immediately before and immediately after each activity. For the Jawbone^™^, it was synced both immediately before and immediately after each individual activity. The ActivPAL^™^ device was programmed before each individual activity, a procedure that cleared all previous data. Additionally, to prevent capturing data pre- or post-activity, all participants were instructed to be/stand still before commencing the activity and to be/stand still once the activity was completed. Participants were also video recorded with a hand-held camcorder during each activity excepting automobile driving and the bus journey when participants were seated for the duration of testing. The timing for each activity, excepting the automobile driving and bus journey, was started at the same time as the video began recording. The step count obtained from each activity monitor was compared to the step count obtained from the recorded video.

### Prescribed activities

Nine typical activities of daily living were prescribed and preformed in a controlled and monitored way. Activities were grouped into three classes, as passive non-stepping activities (deskwork, elevator, bus journey, automobile driving), moderate non-stepping activities (washing and drying dishes, functional reaching task) or active non-stepping activities (indoor cycling, outdoor cycling, indoor rowing).

#### 1. Deskwork

Participants sat at a desk doing deskwork for 40 minutes. The majority of this time was spent typing.

#### 2. Elevator

Participants took an elevator ride, up 3 floors and down 3 floors, three times.

#### 3. Bus journey

Participants took a 3km round trip bus journey while sitting in the middle of the bus. The bus route travelled through a university campus, which featured speed ramps at various intervals.

#### 4. Automobile driving

Participants drove a manual gear car for a total of ~ 50km. The driving route consisted of driving on a local road (~ 2km, driving in a built-up area), urban road (~ 5km, city centre driving), driving on a category A road (~ 6km, national primary road), driving on a category B road (~ 14km, regional road), driving on a category C road (~ 8km, rural country road) and driving on a motorway (~ 15km).

#### 5. Washing and drying dishes

Participants were instructed to wash dishes at a sink while standing still, without taking any steps. Ten pieces of Tupperware were scrubbed three times on the inside and three times on the outside using a dish-washing brush held in the dominant hand. The items were then dried with three strokes on the outside and three strokes on the inside using a dishcloth. This process was repeated continuously for a total of ten minutes.

#### 6. Functional reaching task

Participants stacked 10 books taking them individually from the floor and stacking them on a shelf just above eye level. An adjustable shelf was used and adjusted based on the height of each participant to be just above his or her eye level. All participants had a similar reach effort when carrying out this task. When all books were stacked the procedure was reversed and the books were individually returned to the floor. This action was repeated continuously for ten minutes.

#### 7. Indoor cycling

Participants cycled on a Kettler Cycle Ergometer (Heinz Kettler GmbH & Co., Germany) for a total of 2km at a speed of 15-20km/h set at a comfortable intensity.

#### 8. Outdoor cycling

Participants cycled a pedal bike at a self-selected easy pace for a total of 2km over a tarmacadam surface at a comfortable intensity.

#### 9. Indoor rowing

Participants rowed 2km at a low resistance setting at a stroke rate of 20–25 strokes/minute on a Concept 2 rower (Concept2, Inc. Morrisville, Vermont, US).

### Statistical analysis

All statistical analyses were carried out using SPSS (SPSS for Mac, version 22, IBM Corporation). A negative binomial regression analysis was used to model the step count data with follow-up simple comparison (least significant difference) used to detect differences between the reference category (video recordings) and the false positive steps registered by each activity monitor. A false positive (FP) was taken as a step registered by the monitors when no step was actually taken. The video recordings were used as the criterion measure. As a result of the nature of the study, a high number of 0 steps were observed during some activities. In these cases, specifically deskwork and elevator ride, comparisons were made descriptively.

## Results

**Fig 2:**
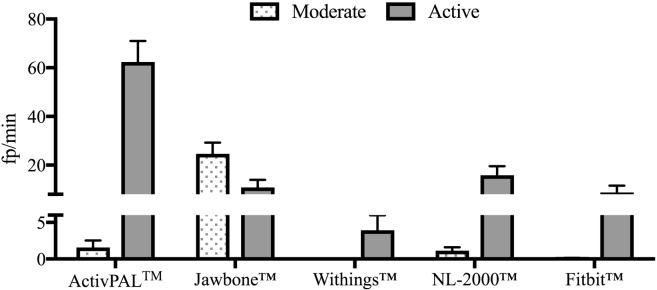
Overall specificity (fp/min) of activity monitors during the moderate and active non-stepping activities. Fp/min = false positives per minute. N = 10/activity. Data represents mean ± SEM.

All monitors tested recorded steps when no steps actually took place (false positives) to a greater or lesser extent depending on the activity being performed. With the exception of the Jawbone^™^ device, all other monitors generated most false positives during active non-stepping activities (Fig 2). The Jawbone^™^ registered its greatest number of false positives during moderate non-stepping activities.

### ActivPAL^™^ activity monitor

**Fig 3:**
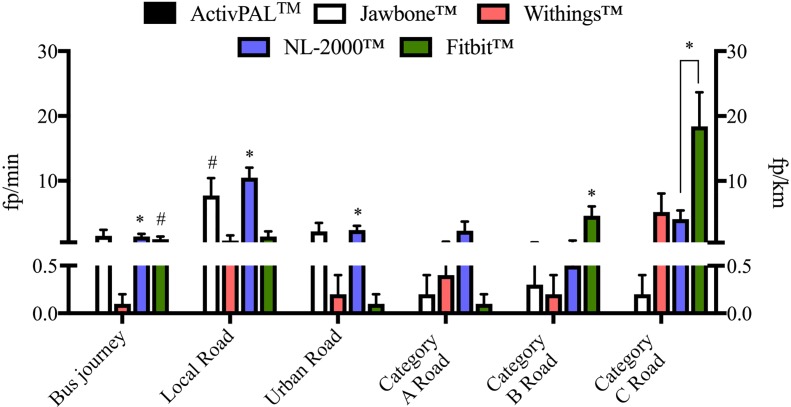
Specificity of each activity monitor during the passive non-stepping activities. Data for deskwork, taking an elevator and automobile driving on a motorway have been excluded due to the high number of zero false positives correctly recorded by the devices during these activities. The mean false positives detected during the bus journey are expressed as false positives detected per minute as represented by the left y-axis. The mean false positives detected during the driving activity are expressed as false positives detected per kilometer driven as represented by the right y-axis. * P ≤ 0.001 vs. zero false positives, # P < 0.05 vs. zero false positives. N = 10/activity. Data represents mean ± SEM.

**Fig 4:**
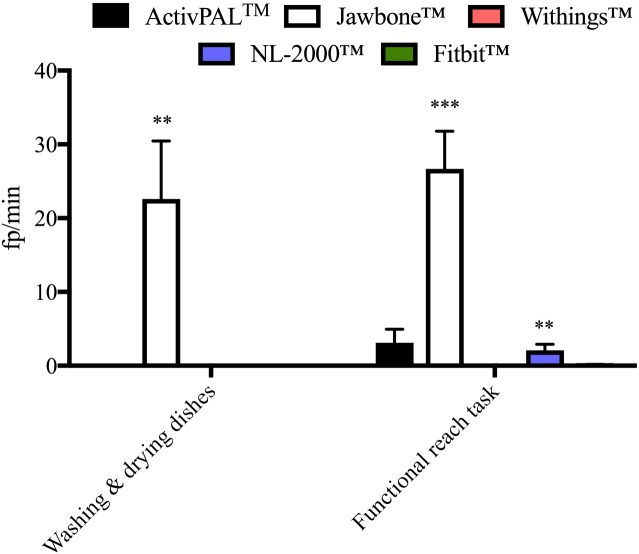
Specificity (fp/min) of each activity monitor during the moderate non-stepping activities. While the ActivPAL^™^ registered a number of fp/min during the functional reaching task, this was found to be non-significant. *** P < 0.001 vs. video recording (zero fp/min), ** P < 0.01 vs. video recording (zero fp/min). N = 10/activity. Data represents mean ± SEM.

**Fig 5:**
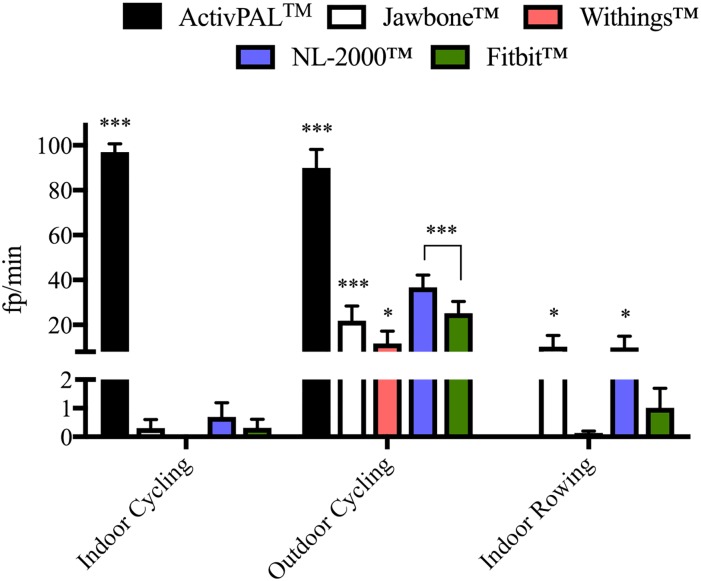
Specificity (fp/min) of each activity monitor during the active non-stepping activities. *** P < 0.001 vs. video recording (zero fp/min), * P < 0.05 vs. video recording (zero fp/min). N = 10/activity. Data represents mean ± SEM.

The ActivPAL^™^ activity monitor was found to be specific for step detection during all but one of the passive and moderate non-stepping activities (Figs 3 and 4). For the functional reaching task the ActivPAL^™^ registered a non-significant number of false positives. The ActivPAL^™^ correctly (no false positives registered), did not record any steps during the other activities: deskwork, taking an elevator ride, taking a bus journey, automobile driving or washing and drying dishes (Table 2). This was also true during the active non-stepping activity of indoor rowing, whereby the ActivPAL^™^ correctly did not register any false positives. However, the ActivPAL^™^ was found to be less specific for step detection during the cycling activities. The ActivPAL^™^ registered 97±12 fp/min during the indoor cycling activity and 90±26 fp/min during the outdoor cycling activity (Fig 5).

**Table 2:** Mean false positives per minute for all activity monitors during the non-stepping prescribed activities.

	ActivPAL^™^		Jawbone^™^		Withings^™^		NL-2000^™^		Fitbit^™^	
	FP ± SD	P Value	FP ± SD	P Value	FP ± SD	P Value	FP ± SD	P Value	FP ± SD	P Value
Desk work (min)	0	—	0	—	0	**—**	0	**—**	0	**—**
3^rd^ floor elevator (min)	0	—	1 ± 2	—	0	**—**	0	**—**	0	**—**
Bus journey (min)	0	—	2 ± 3	0.090	0	0.292	1 ± 1	**0.001**	1 ± 1	**0.012**
Automobile driving: Local road (km)	0	—	8 ± 8	**0.002**	1 ± 2	0.292	10 ± 5	**< 0.001**	1 ± 3	0.062
Automobile driving: Urban road (km)	0	—	2 ± 4	0.080	0 ± 1	0.292	3 ± 2	**< 0.001**	0	0.292
Automobile driving Category A road (km)	0	—	0	0.292	0 ± 1	0.057	2 ± 4	0.088	0	0.292
Automobile driving: Category B road (km)	0	—	0 ± 1	0.138	0 ± 1	0.292	1 ± 1	0.086	5 ± 5	**0.001**
Automobile driving: Category C road (km)	0	—	0 ± 1	0.292	5 ± 9	0.052	4 ± 4	**0.001**	18 ± 17	**< 0.001**
Automobile driving: Motorway (km)	0	—	0	—	0	**—**	0	—	0	—
Washing and drying dishes (min)	0	—	23 ± 25	**0.002**	0	**—**	0	—	0	—
Functional reaching task (min)	3 ± 6	0.064	27 ± 16	**< 0.001**	0	—	2 ± 3	**0.006**	0	0.292
Indoor cycling (min)	97 ± 12	**< 0.001**	0 ± 1	0.495	0	0.136	1 ± 2	0.136	0 ± 1	0.057
Outdoor cycling (min)	90 ± 26	**< 0.001**	22 ± 21	**< 0.001**	12 ± 17	**0.025**	37 ± 17	**< 0.001**	25 ± 17	**< 0.001**
Indoor rowing (min)	0	—	10 ± 16	**0.032**	0	0.292	10 ± 16	**0.033**	1 ± 2	0.123

All false positive values represent mean ± SD. The mean false positives detected during the driving activities have been adjusted to reflect the false positives detected per kilometer driven. This is to allow for direct comparison between the step counts detected on each road type. All other false positive values represent the false positives detected per minute of each activity. Significant values are highlighted in bold. ‘—’ indicates no p value obtained. FP = False Positives; SD = Standard Deviation. N = 10/activity.

### Jawbone^™^ activity monitor

The Jawbone^™^ activity monitor was found to be specific during the majority of the passive non-stepping activities aside from driving on a local road with the Jawbone^™^ registering on average 8±8 fp/km. No statistically significant number of fp/km or fp/min was registered on any other road type or during the remaining passive non-stepping activities of deskwork, taking an elevator ride or taking a bus journey (Table 2). The Jawbone^™^ was found to be less specific during the moderate non-stepping activities where 23±25 fp/min were registered while washing and drying dishes and 27±16 fp/min during the functional reaching task (Fig 4). Similar results were observed during the active non-stepping activities of outdoor cycling (22±21 fp/min) and indoor rowing (10±16 fp/min) but not during indoor cycling (Fig 5).

### Withings^™^ activity monitor

Similar to the ActivPAL^™^ activity monitor, the Withings^™^ monitor was found to be specific during all passive and moderate non-stepping activities (Figs 3 and 4). Although a small number of fp/min were registered for a small number of participants during the automobile driving activity, these values did not reach statistical significance (Table 2). The Withings^™^ was also found to be specific during the active non-stepping activities of indoor cycling and indoor rowing, whereby the participants were active, but on a static exercise machine. The Withings^™^ correctly did not register any steps during these activities. However, this was not true during outdoor cycling. Cycling outside over a standard tarmacadam road surface on a pedal bicycle resulted in the Withings^™^ registering a significant number of false positives, averaging 12±17 fp/min (Fig 5).

### NL-2000^™^ activity monitor

While the NL-2000^™^ activity monitor was found to be specific during the passive non-stepping activities of deskwork and taking an elevator ride, the same results were not observed during the bus journey or while automobile driving. A significant number of false positives were registered during the bus journey and on certain road types during automobile driving. The NL-2000^™^ registered 10±5 fp/km while driving on a local road, 3±2 fp/km driving on an urban road and 4±4 fp/km driving on a category C road, all of which were statistically significant (Fig 3). A number of fp/km were also registered on the NL-2000^™^ while driving on the category A and B roads but these values did not reach statistical significance (Table 2). The NL-2000^™^ was found to be specific while washing and drying dishes but not during the functional reaching task, where 2±3 fp/min were registered (Fig 4). While the NL-2000^™^ registered very few fp/min during the indoor cycling activity, a significant number of fp/min were registered during the outdoor cycling and indoor rowing activities. An average of 37±17 fp/min were registered during outdoor cycling, with an average of 10±16 fp/min registered during indoor rowing (Fig 5).

### Fitbit^™^ activity monitor

Similar to the other monitors examined, the Fitbit^™^ monitor was found to be specific during the deskwork and elevator ride activities, correctly registering zero false positives during these activities (Table 2). However, similar to the NL-2000^™^ monitor, the Fitbit^™^ registered a significant number of false positives during the bus journey and on certain road types during automobile driving (Fig 3). During the automobile driving activity, the Fitbit^™^ registered 5±5 fp/km while driving on a category B road and 18±17 fp/km while driving on a category C road. However, the Fitbit^™^ was found to be specific during the moderate non-stepping activities of washing and drying dishes and during the functional reaching task, correctly registering zero false positives (Fig 4). This was also observed during the active activities of indoor cycling and indoor rowing, but for a small number of participants where a non-significant number of false positives were registered for each activity. However, the Fitbit^™^ was found to register false positives during outdoor cycling, registering a significant average of 25±17 fp/min during this activity (Fig 5).

## Discussion

This study assessed five activity monitors: the Jawbone UP^™^, Withings Smart Activity Monitor Tracker (Pulse O_2_)^™^, NL-2000 pedometer^™^, ActivPAL micro^™^ and Fitbit One^™^ for their specificity for step detection during a series of non-stepping activities, representing examples of typical activities of normal daily living.

While there are many possible outputs from most activity monitors, such as time spent active, distance moved and calories burned, step count remains one of the most popular and translatable outputs in use today. Many research studies and consumer electronic articles have focused on step count as an output for measuring physical activity [11, 15, 16]. Many national and international health organizations have provided recommendations on step count goals in line with improving population health. The current recognized recommendation is 10,000 steps per day, which is a convenient method of physical activity quantification for the general public to comprehend. As many activities of daily living involve both stepping and non-stepping movements, it is important that activity monitors are both sensitive and specific in their detection of their primary output, i.e. actual steps taken. Previously, we have shown a number of physical activity monitors to be sensitive in step detection when actively walking over a variety of surfaces and in different types of footwear, while reaching the recommended daily 10,000 step goal [12]. However, our previous work focused on the performance of the activity monitors during purposeful stepping only. A critical question for activity monitors is, are steps recorded when no steps are actually taken? Considering this issue, in our current study we tested the specificity of activity monitors in distinguishing stepping from non-stepping movements during a number of prescribed activities typical of activities of daily living. It is important to gain an understanding of the true specificity of activity-monitoring devices in situations reflective of normal everyday activities.

In this paper we report on specificity as the number of false positives registered per minute for each activity. Our results demonstrate that, when grouped by activity type, the majority of the activity monitors tested were least specific during active non-stepping activities, which involved indoor cycling, outdoor cycling and indoor rowing. As these activities involve a significant amount of non-stepping body movements, and considering the typical movements involved in these activities, it is not entirely surprising that a number of false positive were registered specifically during these activities. It is interesting to note however, that during the indoor activities on a static rowing or cycling ergometer, there was less variation in the false positive count compared to outdoor cycling. During outdoor cycling, all activity monitors registered a significant number of false positives.

While the ActivPAL^™^ monitor was found to be specific during passive and moderate non-stepping activities, a significant number of false positives were registered during the active cycling activities. This is likely due to the continuous changing of the leg position from the horizontal to the vertical plane and thus the location of the ActivPAL^™^ on the anterior aspect of the thigh has a role to play in these results. In a similar manner, the Jawbone^™^ activity monitor registered the greatest number of false positives during the moderate non-stepping activities of washing and drying dishes and the functional reaching task. This may reflect the fact that the Jawbone^™^ is worn on the wrist. Both moderate non-stepping activities performed focused on upper body movement involving both the arms and hands, which mostly likely played a role in the significant number of false positives registered by the Jawbone^™^. Additionally, the Jawbone^™^ registered a significant number of false positives during automobile driving on a local road. The local road, through a built-up area, consisted of a number of speed bumps and required regular gear changes. As the Jawbone^™^ recommended wearing location is the non-dominant wrist, the majority of participants wore the Jawbone^™^ on the left wrist throughout this study, which is the hand involved in gear changing in the manual car used in this work. This, at least in part, may explain why the Jawbone^™^ registered a significant number of false positives during this section of automobile driving. It could be interesting to assess different placements of the Jawbone^™^ while driving. The Jawbone^™^ was not the only activity monitor found to register a significant number of false positives during automobile driving however. The NL-2000^™^ registered a significant number of false positives on a local road, an urban road and a category C road, while the Fitbit^™^ also registered a significant number of false positives on a category C road in addition to on a category B road. These monitors also registered a significant number of false positives during the bus journey. As the NL-2000^™^ monitor was worn at the waist and on the left had side of the body in this study, which is the clutching side in a manual car, this may have affected the false positives recorded. The Fitbit^™^ however, was worn at the level of the chest, and as with the bus journey, it is likely that surface type, i.e. the bumpiness of the road, played a role in the false positives observed in that case.

These findings are relevant to all users of physical activity monitors from the typical sedentary user to the most active of users. The sensitivity and specificity of these devices are important considerations for both the general public interested in tracking step count for personal achievement, but also special populations tracking activity as part of a lifestyle prescription. Increasingly, wearable activity monitors are part of a suite of tools employed in tackling conditions such as obesity, COPD and type-2 diabetes. In these instances, the activity monitors play a critical role in monitoring progress towards improving physical activity levels and goal setting as part of behavioral change lifestyle interventions.

By way of example we can take four individuals representing a spread of activity levels from sedentary (taxi driver), low active (office worker), somewhat active (school teacher) to highly active (student football player). Based on these classifications individuals would be expected to achieve less than 5000 steps, 6250 steps, 8750 steps or greater than 12500 steps per day on average respectively [11]. The possible effect of false positives generated by each monitor on daily step count is presented in Table 3.

**Table 3:** Estimated false positives generated daily based on conservative estimates of time spent on sample activities for different categories of potential activity monitor users over a typical 12-hour day.

	Taxi Driver (5000 steps/day)					Office Worker (6250 steps/day)					School Teacher (8750 steps/day)					Student Football Player (12000 steps/day)				
	T/D	AM 1	AM 2	AM 3	AM 4	T/D	AM 1	AM 2	AM 3	AM 4	T/D	AM 1	AM 2	AM 3	AM4	T/D	AM 1	AM2	AM3	AM4
Deskwork (min)	-	-	-	-	-	360	0	0	0	0	120	0	0	0	0	120	0	0	0	0
3^rd^ floor elevator (min)	-	-	-	-	-	15	15	0	0	0	5	5	0	0	0	-	-	-	-	-
Bus journey (min)	-	-	-	-	-	60	120	0	60	60	-	-	-	-	-	60	120	0	60	60
Driving: Local road (km)	40	320	40	400	40	-	-	-	-	-	10	80	10	100	10	-	-	-	-	-
Driving: Urban road (km)	35	70	0	105	0	-	-	-	-	-	10	20	0	30	0	-	-	-	-	-
Driving: Category A Road (km)	-	-	-	-	-	-	-	-	-	-	5	0	0	10	0	-	-	-	-	-
Driving: Category B Road (km)	-	-	-	-	-	-	-	-	-	-	5	0	0	5	25	10	0	0	10	50
Driving: Category C Road (km)	-	-	-	-	-	30	0	150	120	540	5	0	25	20	90	10	0	50	40	180
Driving: Motorway (km)	-	-	-	-	-	-	-	-	-	-	10	0	0	0	0	-	-	-	-	-
Washing and drying dishes (min)	20	460	0	0	0	20	460	0	0	0	20	460	0	0	0	20	460	0	0	0
Functional reaching task (min)	15	405	0	30	0	30	810	0	60	0	20	540	0	40	0	60	1620	0	120	0
**Total (false positives)**		1255	40	535	40		1405	150	240	600		1105	35	205	125		2200	50	230	290
**% of the average daily step count that is false**		25.1	0.8	10.7	0.8		22.5	2.4	3.8	9.6		12.6	0.4	2.3	1.4		18.3	0.4	1.9	2.4

Values for the automobile driving activity are expressed as the number of false positives detected per kilometer driven. Values for all other activities are expressed as the number of false positives detected per minute. The ActivPAL^™^ monitor has been excluded from this table, as it correctly did not register any false positives during the included sample activities. T/D = Time in minutes/Distance in kilometers; AM = Activity Monitor. AM 1 = Jawbone^™^; AM 2 = Withings^™^; AM 3 = NL-2000^™^; AM 4 = Fitbit^™^.

As is evident from Table 3, across a spectrum of users from sedentary to highly active, all monitors have the capacity to contribute false positives to daily step counts. A recent survey published by the National Transport Authority of Ireland reported that taxi drivers drive a minimum of 4 and a maximum of 5 hours per day. Within this timeframe taxi drivers drive up to 14.9 kilometers per hiring cycle, with hiring cycles lasting anywhere up to 59 minutes [17]. Based on our conservative estimates in Table 3, the Jawbone^™^ and NL-2000^™^ can generate false positives accounting for up to 25.1% and 10.7% of the daily average of 5000 steps for a sedentary Taxi driver [11]. Had the Taxi driver been using a Withings^™^ or Fitbit^™^ device in our example then the false positives could be significantly less.

In a typical day in the life of an office worker, washing and drying dishes, carrying out several hours of deskwork, stacking books, taking the elevator several times throughout the day, taking a bus and short car journey to and from work is not uncommon. Again, based on our estimates, the Jawbone^™^ monitor can generate false positives accounting for up to 22.5% of the daily average of 6250 steps for a low active office worker. Using a Withings^™^, NL2000^™^ or Fitbit^™^ could have resulted in a much lower contribution of false positives at 2.4, 3.8 and 9.6% of the daily average respectively. A similar pattern is apparent for a typical active user such as a schoolteacher, while a highly active user such as a student football player could equally be exposed to the accumulation of false positive step counts.

While a typical day in the life of the average activity monitor user will be highly variable and could consist of many different stepping and non-stepping activities, in this paper we have highlighted the effect of some normal activities of daily living on step count. Our data, and when considered for typical users in Table 3, serve to highlight the potential for false positive steps to be registered during a normal day consisting of some passive, moderate and active non-stepping activities. In a similar study carried out by Stackpool *et al* [18] participants wore the Nike Fuelband, Fitbit Ultra, Jawbone UP and NL-2000i pedometer when utilising an elliptical cross-trainer and carrying out agility exercises in a gymnasium, including agility ladder drills (seven different moves), 10 basketball free throws and a basketball half-court lay-up drill for one minute. The authors reported inaccuracies in the activity monitors during the agility tests, with all monitors excepting the Jawbone UP significantly underestimating steps [18]. The Nike Fuelband was found to underestimate steps by 34%, the Fitbit Ultra by 20% and the NL-2000i by 17%. During the elliptical exercise the only significant difference lay with the NL-2000i, which underestimated steps by 6% [18]. Although these monitors underestimated step counts during these activities and our results highlight the potential for the registering of false positive steps, both studies highlight the variability that can occur during physical activities that are not specifically stepping activities.

Overall, this data is important to consider when utilising activity monitors in both the research and clinical domains whether using research grade or off-the-shelf consumer-based tracking tools. While the ActivPAL^™^ is a research-grade activity monitor and the other activity monitors tested here are consumer-grade monitors, the specificity was observed to vary for all monitors during one or more non-stepping activity. However, all activities in this study are classed as physical activity and go towards maintaining a healthy and active lifestyle. Accurate translation of the activity carried out during these non-stepping activities into step count could be of interest. Clearly it is important to consider non-stepping activities when wearing physical activity monitors to accurately quantify overall physical activity levels. It is evident from our data that typical non-stepping activities, likely to be carried out in everyday life, result in the recording of steps when none are taken. As mentioned above, these factors are especially important to consider in situations whereby physical activity is prescribed as an intervention in the prevention or treatment of chronic disease, where accurate physical activity quantification is of the utmost importance.

## Conclusions

This study provides the first comprehensive specificity evaluation of five activity monitors during a variety of prescribed physical activities involving non-stepping body movements. From our results, we can conclude that false positive steps are detected and registered during a range of non-stepping activities, affecting the quantification of physical activity with regard to step count as the primary output. The range and impact of the false positives detected will differ depending on the activities carried out and the activity monitors utilised, with the results highlighting the specificity variability that can exist during a range of non-stepping physical activities. The Withings^™^ monitor performed best across all non-stepping activities, registering a significant number of fp/min only during the outdoor cycling activity. The specificity of an activity monitor needs to be considered carefully when choosing an activity monitor to accurately quantify the variety of physical activities that can be carried out on any typical day.
